# The effects of early administration of atropine during dobutamine stress echocardiography: advantages and disadvantages of early dobutamine-atropine protocol

**DOI:** 10.1186/1476-7120-4-17

**Published:** 2006-03-29

**Authors:** Ana Cristina Camarozano, Aristarco G Siqueira-Filho, Luis Henrique Weitzel, Plínio Resende, Rosângela Aparecida Noé

**Affiliations:** 1 Cardiology Department, Barra D'or Hospital, Rio de Janeiro, Brazil; 2 National Heart Institute, Rio de Janeiro, Brazil; 3 Internal Medicine Department, University Hospital, Federal University of Rio de Janeiro (UFRJ), Rio de, Janeiro, Brazil; 4 Cardiology Department, National Heart Institute, Rio de Janeiro, Brazil; 5 Cardiology Department, Barra D'or Hospital, Federal University of Rio de Janeiro, Rio de Janeiro, Brazil; 6 Statistical Department, Federal University of Rio de Janeiro, Rio de Janeiro, Brazil

## Abstract

**Background:**

The conventional dobutamine protocol for the investigation of induced myocardial ischemia is well established. Our objective was to evaluate the effects of early administration of atropine during the dobutamine stress echocardiogram, as compared to its conventional use.

**Methods:**

One hundred and twenty-one patients were referred to the dobutamine stress echocardiogram, for the investigation of myocardial ischemia and the administration of atropine was randomized into three groups (A, B, C at 10, 20 and 40 mcg/kg/min of dobutamine, respectively).

**Results:**

The mean level of the double product was significantly lower in the group C patients when compared to group B patients (p = 0.002). The mean test time (12.8 ± 3.1 and 18.7 ± 3.4 p= 0.0001) and the mean total dose of dobutamine (14 × 18 × 25 mg p = 0.008) were significantly higher in group C patients than in group A & B patients. The mean test time was reduced in 6 minutes (31%) with the early administration of atropine in relation to the standard protocol. The atropine dose used in the different groups was similar. Complications were uniform in all cases.

**Conclusion:**

The early administration of atropine during the dobutamine-atropine stress echocardiography significantly reduces duration of the test and the dose of amine without increasing the number of complications, the total dose of atropine or the number of diagnostic tests.

## Background

Dobutamine-atropine stress echocardiography (DASE) is well established in clinical practice, as it is considered to be one of the main methods of imaging to determine the presence of myocardial ischemia.

Currently, the use of the 3 min. protocol has become the most popular, beginning at 5 mcg/kg/min infusion of dobutamine and reaching a maximum dose of 40 mcg/kg/min, with the addition of atropine from the final stage on. Even though atropine has been used more often at the end of the stress test to increase heart rate and accuracy, this agent is usually administered during or soon after the maximum dose of dobutamine, with the patients receiving prolonged amine infusions which may increase side effects. Besides, the test time is often extended.

Considering that a significant number of patients (32%) do not reach a dobutamine stress echocardiographic end point with the standard protocol [1], and the increasing use of Beta blockers as anti-ischemic or anti-hypertensive medication in the last decade, the following question should be considered: Are the protocols employed in stress echocardiography still in agreement with current medical practice?

If the range of the ideal heart rate, which is of extreme importance to the accuracy of the method, is compromised, then the need for a more homogenous protocol which counterbalances the employed therapeutic effect becomes relevant so as to avoid damage to the diagnostic and prognostic information of the test.

The aim of this study was to evaluate the effects of atropine when initiated early during dobutamine stress echocardiography in relation to its later usage.

## Methods

One hundred and twenty-one consecutive patients examined were submitted to dobutamine stress echocardiography. Treatment with anti-ischemic medication was not interrupted before the test.

**Figure 1:**
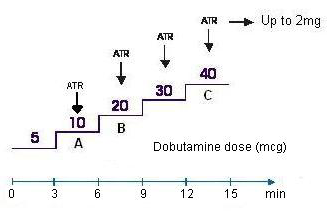
Protocols A, B and C.

The patients were randomly divided into three protocols of study (Figure 1): 1A) early administration of atropine at a dose of 10 mcg/kg/min of dobutamine; B) early administration of atropine at a dose of 20 mcg/kg/min of dobutamine, and; C) late or standard administration of atropine at a dose of 40 mcg/kg/min of dobutamine, in case heart rate obtained was below 85% of maximum rate expected for age (220 – age) and there was no criteria for interruption of test (such as: new abnormality in wall motion or a worsening of an existing one, heart rate = or > 85% of maximum rate expected for age, important increase of systolic or diastolic blood pressure (SBP > 220 mmHg and/or DBP > 110 mmHg), relevant cardiac arrhythmia (supraventricular arrhythmia with high response and/or malignant ventricular arrhythmia), intense angina (specially if associated with worsening of regional function), pronounced hypotension (decrease of SBP > 20 mmHg) followed by symptomatology, and conclusion of protocol. Groups A & B were considered belonging to the early dobutamine-atropine protocol (Figure 1) and group C belonged to the late protocol (conventional).

In group C, some patients did not require atropine during the test (which is part of the standard protocol). The patients of group C who did not receive atropine during the test (n = 16) were not taken into account for pertinent statistical analysis on the effects of atropine in the early or late phase of the echocardiogram. For a better understanding, we considered to be late protocol the patients of group C who received atropine and standard protocol the patients involved in group C who did or not require atropine.

The current research was approved by the Ethical and Research Committee of the hospital, and informal consent was obtained from all the patients.

### Stress echocardiogram

Two kinds of equipments were used in the study: a HDI 5000 (digitalization – "Image View") and a Sonos HP 5500, both with 4,0 MHz transducers.

The patients were submitted to the two-dimensional echocardiograms and Doppler for full analysis of cardiac performance. The images obtained were of the long axis, short axis, apical four and two chambers which correspond to the viewing of the myocardial segments supplied by the main coronary arteries.

Dobutamine was diluted in a glucose solution and administered through an infusion pump initially at a dose of 5 mcg/kg/min and increased to 10, 20, 30 mcg/kg/min every 3 minutes up to maximum dose of 40 mcg/kg/min (according to each group). The atropine sulfate was initiated at a dose of 0,25 up to 0,50 mg and increased in intervals of 1 to 2 minutes respectively, until a maximum dose of 2 mg. This agent was administered intravenously in the last minute of the stage, whereas the dobutamine infusion remained continuous.

During the exam, a three derivation electrocardiogram (ECG) record was maintained, as well as a continuous check up of heart rate and blood pressure before and after the administration of atropine.

At the peak of test, after achieving the end points, the dobutamine and atropine infusions were interrupted and monitoring was continued during 5 to 10 minutes of the recovery phase (eventually, for a longer period if necessary), until heart rate was lower than 100 bpm and other clinical and hemodynamic parameters were normal. Metoprolol was given intravenously (1 to 5 mg) during recovery phase to revert the effects of the dobutamine-atropine combination.

### Echocardiographic evaluation

The left ventricle was divided into 16 segments [2]. Each segment was described as: normal, hypokinetic, akinetic or dyskinetic. A normal dobutamine stress echocardiogram was defined as having normal wall motion at rest with an increase of its systolic thickening during stress (hyperkinesia). The presence of ischemia (positive test) was defined as the development of a new abnormality in wall-motion or a worsening of an existing one during stress (hypokinesia, akinesia or dyskinesia).

The echocardiograms were reviewed by two experienced examiners ("off line"), who had no access to clinical and angiographical data, and a consensus was reached whenever necessary.

### Statistical analysis

The statistical methodology adopted for the comparison of proportions between continuous variables was the *X^2 ^test *or the *Fisher exact Test*, whenever the *X^2 ^test *could not be evaluated. For the comparison of means between the two groups the *t de Student test *was applied for independent samples, or the *Mann-Whitney test *(non-parametric test) whenever the variable did not present normal distribution.

In situations where three groups had to be analyzed, the comparison of means between the groups was made with the *Analysis of Variance *(ANOVA). In this case, the *Tukey *test of multiple comparisons was applied to identify which groups differed from each other; this is an ANOVA complementary test. The *Analysis of Variance of Kruskal-Wallis *(non-parametric test) was used for the comparative analysis of variables which did not present a normal distribution.

The adopted criteria for determining any significance was the level of 5% (in other words, whenever the *p *value of the statistical analysis was lower or equal to 0,05).

The analysis of all statistical tests was processed by the SAS^® ^system (Statistical Analysis System).

## Results

Of the total sample (n = 121), 76 patients (63%) received early atropine in two different groups (A & B), and 45 patients (37%) did or not receive atropine in the late protocol. On average 49% of the patients were taking Beta blockers.

The demographic data of the three Groups are reported in Table 1.

**Table 1:** Demographic characteristics; risk factors; use of medication; and ECG, ECHO and CAT data, in each group.

Variables	Group A (n = 41)	Group B (n = 35)	Group C (n = 29)	*p value*
Age (± DP)	60,8 +/- 11	59,6 +/- 12	54 +/- 12	0,038
Weight	69,2	71,6	74	0,4
Gender, Male %	51,2	54,2	65	0,47
Test indications:				
CAD investigation or pre-operative %	46	66	55	0,23
Evaluation of known CAD %	54	34	45	0,23
Previous history of MI %	40	26	32	0,43
History of angina %	58	68	41	0,089
History of heart failure %	39	21	24	0,17
Hypertension %	61	62	45	0,61
Diabetes Mellitus %	20	20	21	0,98
High cholesterol %	46	62	35	0,1
Family history for CAD %	49	60	46	0,49
Smoking %	29	31	24	0,8
Obesity %	36	28	45	0,4
Use of Beta blockers %	49	54	45	0,74
Use of calcium channel blockers %	12	20	10	0,48
Use of digitalic %	0	6	10	fp
Altered baseline ECG %	38	39	41	0,97
Altered baseline ECHO %	41	37	42	0,87
Normal ventricular function %	66	86	65	0,097
Previous CAT %	46	40	36	0,68
Previous PTCA %	19	12	10	0,6
Previous CABG %	17	17	10	0,69

CAD = coronary artery disease MI = myocardial infarction

PTca = percutaneous transluminal coronary angioplasty CABG = cardiac artery bypass grafting

ECG = eletrocardiogram with 12 derivations ECHO = echocardiogram CAT = cardiac catheterism fp = few patients (<10)

**Figure 2:**
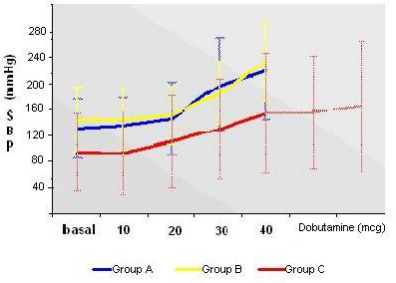
The hemodynamic response (systolic blood pressure) in the three Groups.

**Figure 3:**
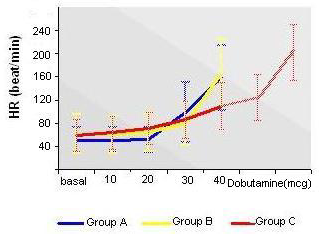
The hemodynamic response (heart rate) in the three Groups.

The hemodynamic response in the three Groups is summarized in Figure 2 (blood pressure) and Figure 3 (heart rate).

**Figure 4:**
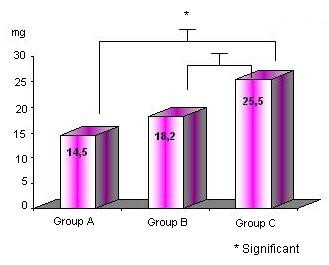
Dobutamine dose in Groups A, B and C*

**Figure 5:**
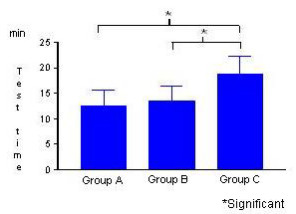
Test time.

The dobutamine dose was lower for Groups A and B (Figure 4) and the test time was shorter for Groups A and B (Figure 5).

**Figure 6:**
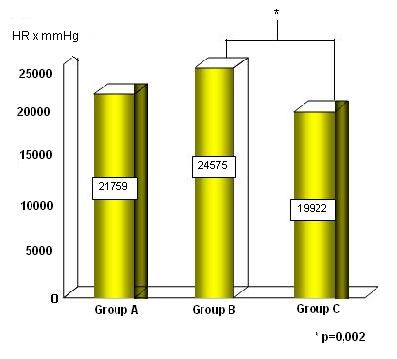
The double product in the Groups A, B and C.

The double product is higher in the Group B, as is showed in Figure 6.

Results and complications of the three Groups are described in detail in Table 2.

**Table 2:** Results and complications of the tests in the three groups.

Variables	Groups			*p value*
	A	B	C	
**Results (%) :**				
Positive	24	17	14	0,44
Negative	54	71	62	
Inconclusive (negative submaximum)	22	11	24	
**Complications (%) :**				
Hypertension	14	14	7	0,61
Supraventricular arrhythmia	10	8,5	3	fp
Ventricular arrhythmia	27	8,5	21	0,12
Hypotension	0	0	10	fp
Chest pain	7	8,5	14	0,7
Others *	17	26	14	0,44
**General Complications**	58,5	54	55	0,92

fp = few patients (<5) * others = nausea, vomiting, tremor, headache

## Discussion

The present study shows that the early administration of atropine during dobutamine stress echocardiogram significantly reduces test time and the dose of the sympaticomimetic amine without, however, increasing the number of general or specific complications. Besides, the total dose of atropine used was not higher when administered early, but the double product presented a significant increase with the addition of this agent when compared to the isolated effect of dobutamine. In this way, the early intervention with atropine resulted in a more balanced test when related to chronotropism and inotropism, maximizing the double product, optimizing time and not increasing side effects. This information is emphasized when we consider the important sympathic-parasympathic interaction in regulating the heart rate and ventricular function[3].

Lessick et al [4] also confirmed our findings, showing a reduction of test time with no increase of side effects caused by the early administration of atropine.

Regarding complications and test results (less number of the sub-maximum tests), group B patients appeared to be more adequate in relation to the other groups, but without statistical significance.

In addition, no patients had any intoxication problem with the higher doses of anti-muscarinic (2 mg), even when administered to elderly patients.

Our findings confirm and expand previous studies suggesting atropine is often required to achieve a maximum hemodynamic stress with dobutamine [5-8] and that earlier administration of atropine does not seem to increase the rate of serious adverse events [5,6,9,10].

### Limitations

Diagnosis of the echocardiogram exams for the presence of parietal disinergy was obtained by the qualitative and subjective techniques, and not through quantitation method of precision. However, visual evaluation of cardiac motion remains not only the simplest method, but also the most accurate for regional analysis [11].

We must also mention that the total testing time calculated only included the duration of the dobutamine infusion and not recovery phase. The reasons that led to this decision were the subjective nature of defining the end of this phase, the custom of administering the antidote (metoprolol) to almost every patient in order to reverse the tachycardia induced by the method, and also due to the fact that the patient may be transferred to another room until all hemodynamic parameters are fully normal, without necessarily using the examination room.

## Conclusion

Based on our findings, we may conclude that:

The early intervention with atropine allowed for a more balanced test regarding chronotropism and inotropism, optimizing time and maximizing the double product, besides being responsible for the reduction of the total dobutamine dose without increasing side effects. In our study, the early administration of atropine at 20 mcg of dobutamine seemed the most adequate since it presented a higher double product and fewer complications and sub-maximum tests. Overall, the early administration of atropine is indicated to all patients submitted to the test, including those patients receiving Beta blockers.

## Competing interests

The author(s) declare that they have no competing interests.
